# Opportunities and Challenges in Using National EHR Networks for AI in Learning Health Systems

**DOI:** 10.1002/lrh2.70090

**Published:** 2026-05-27

**Authors:** Polina V. Kukhareva, Ramkiran Gouripeddi, Niels Peek, Kensaku Kawamoto

**Affiliations:** ^1^ Department of Biomedical Informatics University of Utah Salt Lake City Utah USA; ^2^ THIS Institute (The Healthcare Improvement Studies Institute), Department of Public Health and Primary Care University of Cambridge Cambridge UK

**Keywords:** artificial intelligence, data harmonization, electronic health records, health data integration, learning health systems, National Electronic Health Record Networks

## Abstract

**Background:**

National electronic health record (EHR) networks can support learning health systems (LHSs) by enabling large‐scale data aggregation, monitoring, and benchmarking, but their capacity to produce trustworthy and locally deployable machine learning and artificial intelligence (ML/AI) models remains uncertain. We characterized major US national EHR networks and examined barriers to ML/AI development and deployment across the LHS cycle.

**Methods:**

We conducted an environmental scan combining PubMed searches with reviews of network websites, governance documents, and federal and vendor white papers through September 2025. Eligible networks aggregated patient‐level EHR data nationally. We abstracted scale, settings, data domains, harmonization approaches, and access models, identified ML/AI studies, and mapped barriers onto a seven‐step LHS‐AI cycle from data capture to implementation and monitoring.

**Results:**

We identified 23 national EHR networks spanning federal and academic consortia, vendor‐led consortia, commercial aggregators, and practice‐based research networks, covering fewer than 1 million to more than 200 million patients. Most used common data models to standardize inputs. We identified 34 ML/AI studies, but only a small subset was prospectively evaluated or integrated into clinical workflows. Common barriers included heterogeneous data capture, privacy and linkage constraints, residual variation despite harmonization, limited representativeness, need for local recalibration, and sociotechnical challenges related to implementation and evaluation.

**Conclusion:**

National EHR networks offer critical infrastructure for LHSs but currently function primarily as research rather than ML/AI platforms. Addressing data, implementation, and evaluation barriers to enable ML/AI development and subsequent local deployment is essential to realizing their potential.

## 1 Background

Learning health systems (LHSs) represent a paradigm in which science, informatics, incentives, and organizational culture align to support continuous improvement in health care delivery [1]. Central to this concept is a closed feedback loop in which data generated during routine care are analyzed to produce new knowledge, which is then acted upon to change clinical practice. For example, an LHS can systematically compare artificial intelligence (AI) model predictions and clinician predictions against actual patient outcomes at subsequent encounters, identify discrepancies, and revise care protocols and algorithms accordingly. National‐scale electronic health record (EHR) data support key LHS activities, including descriptive epidemiology, identification of emerging or persistent problems at the national level, benchmarking regional performance, assessing regional variation in case mix, assessing heterogeneity of treatment effects, and informing whether interventions are likely to generalize across geographies [2, 3, 4]. However, as we describe in this paper, the current national EHR infrastructure has not yet demonstrated the capacity to fully close this learning loop.

Computable biomedical knowledge (CBK) includes machine‐interpretable artifacts such as predictive models, clinical guidelines, and clinical decision support algorithms that translate data into actionable insights at the point of care [5]. Within this landscape, machine learning and AI (ML/AI) models represent one type of CBK that can support LHS activities by enabling earlier intervention, prioritizing outreach under resource constraints, reducing clinician burden through automated case finding, and supporting continuous learning through iterative refinement.

National EHR networks represent a promising infrastructure for ML/AI development by aggregating patient‐level data across institutions, harmonizing them, and providing secure analytic environments [6]. However, most were designed primarily for health services research rather than ML/AI development [7], and their suitability to generate generalizable, trustworthy, and locally adaptable models remains unknown. Barriers potentially span data capture, harmonization, governance, model development, local adaptation, and ongoing evaluation.

Our objective was to identify and characterize major national EHR data networks in the United States, describe their governance, coverage, and applications, and examine their capacity to support the full range of activities required to develop, deploy, and iteratively refine ML/AI models within clinical workflows as part of a functioning LHS. By doing so, we highlight both the breadth of these networks and the barriers that must be addressed to realize their potential for national ML/AI algorithm development. To our knowledge, this study provides the first systematic mapping of national EHR networks onto a complete LHS‐AI lifecycle, enabling identification of barriers across all stages from data capture to implementation and continuous learning.

## 2 Methods

### 2.1 Study Design

We conducted an environmental scan of US national EHR data networks to describe their structure, governance, data coverage, and capacity to support ML/AI modeling, as well as to identify related barriers. The scan combined a systematic search of peer‐reviewed literature indexed in PubMed with a structured review of publicly available resources, including official network websites, governance documents, federal program repositories, and vendor white papers. Using predefined inclusion and exclusion criteria, we first identified eligible national EHR frameworks and abstracted information on their size, scope, and applications. We then identified published ML/AI algorithms developed using these frameworks and synthesized reported barriers encountered during their development and implementation.

### 2.2 Data Sources

Our scan included peer‐reviewed literature indexed in PubMed, government websites, and white papers from federal agencies, vendors, and health system coalitions. The complete PubMed queries are provided in the Supporting Information Appendix.

### 2.3 Data Extraction for National EHR Networks

We focused on active, US‐based networks that aggregate EHR data at a national scale and support broad health services research. Aggregation could be distributed or centralized. National scale was defined as participation from all four US Census regions (South, West, Midwest, Northeast). Networks were included if they (1) aggregated patient‐level EHR data; (2) operated nationally in the United States; (3) were active in 2025; and (4) supported broad health services research.

We excluded initiatives that did not meet these criteria. Specifically, we removed (1) inactive or discontinued efforts (e.g., Community Health Applied Research Network [CHARN] [8]); (2) local, single‐system, or regional consortia lacking national reach (e.g., MedStar Health, Utah Population Database [UPDB]); (3) informatics platforms, infrastructures, or standards communities that enable interoperability but are not national EHR databases themselves (e.g., Electronic Medical Records and Genomics Network [eMERGE] [9], Strategic Health IT Advanced Research Projects‐Natural Language Processing [SHARPn] [9], Nationwide Health Information Network [NHIN] [10], National Institutes of Health Biomedical Translational Research Information System [NIH BTRIS] [11], Observational Health Data Sciences and Informatics [OHDSI] [12], Informatics for Integrating Biology and the Bedside [i2b2]) [12]; (4) specialty registries (e.g., Intelligent Research in Sight [IRIS] [13], National Anesthesia Clinical Outcomes Registry [NACOR] [14], National Dental PBRN) [15]; (5) clinical trials networks without a national EHR repository (e.g., Prevention and Early Treatment of Acute Lung Injury [PETAL]); (6) studies based on claims or other non‐EHR sources (e.g., Premier/Provider Intelligence in Clinical Analytics [PINC AI], National Death Index [NDI]); and (7) international datasets outside the United States (e.g., UK Biobank and Clinical Practice Research Datalink/The Health Improvement Network [CPRD/THIN], Medical Informatics in Research and Care in University Medicine [MIRACUM] in Germany, Taiwan Sentinel).

For each eligible network, one reviewer (P.V.K) abstracted information on (1) number of US patients (alive and deceased), (2) primary types of participating organizations, (3) available data domains (e.g., clinical, claims, surveys, genomics), (4) governance and access models, (5) use of data models for harmonization, and (6) year of creation. Patient and organizational counts were verified against the most recent official network websites when available. When discrepancies arose between publications and organizational sources, the latest publicly reported figures were used. All data are reported as of September 2025.

### 2.4 Data Extraction for ML/AI Algorithms

We included algorithms that explicitly used one or more of the identified national EHR frameworks. For each algorithm, we recorded the clinical domain, the network used, and whether the model was internally validated, externally validated across sites, prospectively tested, or reported as deployed (acknowledging that some deployments may have occurred after publication).

We identified reported barriers that limit the development and translation of these models into practice. We operationalize the LHS‐AI cycle as a seven‐stage framework to systematically evaluate barriers across national EHR infrastructures. Specifically, we mapped these barriers to a lifecycle that traces how data move from local health systems into national repositories and return as AI‐enabled decision support: (1) clinical data capture during care delivery, (2) privacy protection, (3) harmonization, (4) aggregation in centralized or federated repositories, (5) model development from national datasets, (6) adaptation for local populations and workflows, and (7) implementation and operation of ML/AI algorithms to close the learning loop [2, 16]. We also incorporated barriers identified from our collective experience [17, 18, 19, 20].

## 3 Results

### 3.1 National EHR Networks

Our PubMed search identified 304 potentially relevant articles. After screening, 162 manuscripts were excluded, leaving 142 papers that described 17 national EHR networks. Additional web searches identified 6 more networks. Across these 23 networks (Table 1), population coverage ranged from under 1 million (All of Us Research Program) to more than 200 million patients (Epic Cosmos). The networks were grouped into four broad categories: (1) federal and academic consortia, (2) vendor‐led consortia, (3) commercial aggregators, and (4) practice‐based research networks (PBRNs).

**TABLE 1:** Federal and academic EHR networks.

Network	N patients	Setting focus	Key data	Access model	Year	Type
All of Us	0.7 M+	National volunteer cohort	EHR, surveys, biospecimens, genomics, wearables	Public data browser; de‐ID data in cloud Workbench	2018	Federal or academic
Allscripts/Veradigm	48 M+	Independent practices and large group primary care and specialties outpatient clinics	Ambulatory EHR data	Data is leveraged by Veradigm (Allscripts) for real‐world evidence services. Research access via data licensing or sponsored studies.	2018	EHR vendor
Athenahealth	50 M+	Ambulatory/primary care, FQHCs	Ambulatory EHR data	Commercial; licensed for research (e.g., HD4A)	2017	EHR vendor
DARTNet	5 M+	Primarily primary care practices, some specialty clinics	Federated EHR data	Federated network coordinated by the DARTNet Institute	2012	PBRN
ENACT	142 M+	Academic medical centers	Federated EHR query system	NIH‐funded; self‐service queries for registered researchers	2023	Federal or academic
ENRGY	3 M+	Rheumatology specialty practices	EHR‐based clinical and outcomes data on rheumatology patients	Membership/participation through the ENRGY PBRN; data sharing governed by the network for research studies	2021	PBRN
Epic Cosmos	270 M+	Epic hospitals and clinics	Limited EHR dataset	Researchers at participating Epic health systems can query aggregate data via Epic tools (e.g., SlicerDicer); detailed line‐level data access requires Epic certification and enclave environment	2020	EHR vendor
Flatiron health research database	3 M+	Oncology specialty: community oncology practices and cancer centers	EHR data (oncology focus): cancer diagnoses, pathology, treatments, outcomes	Commercial/partnership model—Flatiron (Roche) licenses de‐identified oncology data to life science companies and academic collaborators.	2012	Commercial
HCSRN	18 M+	HMOs	EHR + claims in federated CDM	Queries via member institutions	1994	PBRN
IHS NDW	2 M+	IHS and tribal facilities	EHR + admin data	Federally managed; research via IHS/tribal approval	2006	Federal or academic
IQVIA Ambulatory EMR‐US	70 M+	Ambulatory physician offices from community clinics and primary care	Outpatient EMR records	Commercial dataset sold by IQVIA for research. Deidentified EMR data can be linked with claims and other IQVIA data assets	2006	Commercial
Merative (Explorys + MarketScan)	60 M+	Hospitals and primary care	EHR + claims	For‐profit; subscription access to data and analytics	2022	Commercial
N3C	22 M+	Hospitals and academic centers	Centralized EHR (OMOP CDM)	NIH secure enclave; de‐ID data for registered researchers	2020	Federal or academic
Optum	110 M+	Large integrated delivery networks, multispecialty practices, small groups, physician offices, and hospitals	EHR	Commercial RWD; licensed to researchers/industry	2007	Commercial
Oracle/Cerner	100 M+	Cerner hospitals and clinics	EHR	Vendor‐facilitated; data shared with participants and partners	2019	EHR vendor
OSCER	0.2 M+	Community oncology practices	EHR‐derived oncology data	Managed by McKesson/US oncology research; data aggregated centrally, deidentified and available to research partners under agreements	2004	Commercial
PCORnet	80 M+	Academic and CHCs	Distributed EHR data from CRNs	Queries via PCORnet Front Door; approvals required	2013	Federal or academic
PRIME registry	42 M+	National family medicine practices (primary care, rural and urban)	EHR data from certified family physicians	Centralized registry; participating practices allow data flow to ABFM for reporting and research; deidentified data accessible to approved investigators	2016	Federal or academic
Sentinel (FDA)	300 M+	National distributed drug safety network	Claims + some EHR in CDM	FDA‐run; queries via data partners	2016	Federal or academic
TriNetX	110 M+	Hospitals and academic centers	EHR + claims in federated CDM	Commercial platform; cohort queries and de‐ID data via agreements	2013	Commercial
Truveta	120 M+	Large hospital networks	EHR (incl. clinical narratives)	For‐profit; subscription access to data and analytics	2020	Commercial
VA CDW	24 M+	Veterans Health Administration	VA EHRs (VistA and Cerner)	Secure VA‐only access via VINCI	2007	Federal or academic
Vestrum Health	3 M+	Ophthalmology/retina specialty practices	Ophthalmology/retina practices	De‐identified EHR data from electronic medical record systems used by ophthalmology practices	2013	PBRN

Abbreviations: ABFM, American Board of Family Medicine; All of Us, National Institutes of Health All of Us Research Program; CDM, Common Data Model; CHC, Community Health Center; CRN, clinical research network; CRWD, Cerner Real‐World Data (now part of Oracle Health); De‐ID, deidentified (data stripped of direct personal identifiers); EDW, enterprise data warehouse; EHR, electronic health record; ENACT, Evolve to Next‐Gen Accrual to Clinical Trials; ENRGY, electronic rheumatology network for research and quality; FDA, US Food and Drug Administration; FQHC, Federally Qualified Health Center; HCSRN, Healthcare Systems Research Network; HD4A, Health Data for Action; HMO, Health Maintenance Organization; IHS NDW, Indian Health Service National Data Warehouse; IHS, Indian Health Service; IQVIA, IQVIA Holdings Inc., a global health information and technology company; N3C, National COVID Cohort Collaborative; NIH, National Institutes of Health; OMOP, observational medical outcomes partnership; OSCER, Oncology Supply Chain Enterprise Repository (McKesson/US Oncology Research); PBRN, practice‐based research network; PCORnet, National Patient‐Centered Clinical Research Network; PINC AI, Premier/Provider Intelligence in Clinical Analytics; PPRN, patient‐powered research network; PRIME Registry, Primary Care Registry managed by the American Board of Family Medicine; RWD, real‐world data; VA CDW, Veterans Affairs Corporate Data Warehouse; VA, US Department of Veterans Affairs; VINCI, Veterans Informatics and Computing Infrastructure.

Most national EHR networks provide de‐identified or limited datasets to external researchers. Identifiable data typically remain either at the contributing institution or within the EHR vendor's client environment. Several networks also provide access through secure analytic enclaves equipped with computational resources, allowing investigators to analyze large‐scale de‐identified or limited datasets without exporting patient‐level data. Some networks are better in deduplication than others. For example, Patient‐Centered Clinical Research Network (PCORnet) and National COVID Cohort Collaborative (N3C) have deduplication using Datavant, and Epic Cosmos is using their proprietary deduplication algorithm [21].

#### 3.1.1 Federal or Academic Consortia

The United States has developed federally supported and academic consortia that form the backbone of national EHR research. National PCORnet, established in 2014, is a distributed research network that standardizes EHR and linked claims data across Clinical Research Networks (CRNs) and Patient‐Powered Research Networks (PPRNs) using the PCORnet Common Data Model (CDM), enabling interoperable, multisite analyses across more than 47 million patients annually across 50 states in the United States [22, 23]. PCORnet CRNs are largely non‐overlapping and comprise eight networks: Accelerating Data Value Across a National Community Health Center Network (ADVANCE); the Greater Plains Collaborative (GPC); INSIGHT CRN; OneFlorida+; the PaTH Network; the Pediatric Learning Health System (PEDSnet); the Research Action for Health Network (REACHnet); and the Science, Technology, and Research (STAR) CRN.

The N3C, launched in 2020, harmonizes EHR data from over 80 institutions into the OMOP CDM and has become a leading platform for large‐scale ML [2].

The ENACT, launched in 2023, builds on i2b2/SHRINE to enable multisite cohort discovery across 142 million patients and secure enclaves for ML/AI development [24, 25]. ENACT is supported by an NCATS grant. ENACT operates using enclaves, allowing NLP and more detailed analysis, with additional AI analyses enabled through UCSD. The All of Us Research Program, launched in 2018, integrates EHRs, surveys, biospecimens, and genomics from over one million participants with a focus on diversity and health equity [26]. The FDA Sentinel System integrates EHR, claims, and registry data from over 300 million covered lives to monitor medical product safety [27, 28].

Complementing these consortia are federal health system repositories such as the VA Corporate Data Warehouse (24 million veterans) and the Indian Health Service (IHS) National Data Warehouse (2 million American Indian and Alaska Native individuals) [29, 30]. Collectively, these initiatives provide longitudinal, diverse data and standardized CDMs that support predictive modeling and equitable research at national scale.

#### 3.1.2 Vendor‐Led Consortia

Beginning in the 2010s, EHR vendors built national consortia to aggregate client health system data for real‐world evidence, predictive modeling, and population health analytics, though access and governance remain vendor controlled. Epic Cosmos is the largest, with more than 200 million US patient records from over 310 health systems [31]. It supports real‐world evidence and predictive modeling with integrated tools such as SlicerDicer for interactive cohort exploration. Oracle Health Learning Health Network integrates de‐identified longitudinal data from more than 100 hospitals and clinics for research, clinical trial recruitment, and predictive modeling [32]. Veradigm (formerly Allscripts) curates de‐identified ambulatory care EHR data from independent practices and specialty outpatient clinics covering over 100 million patients. Athenahealth aggregates de‐identified ambulatory records from thousands of outpatient clinics, including Federally Qualified Health Centers, supporting large‐scale health services and primary care research.

#### 3.1.3 Commercial EHR Aggregators

Commercial aggregators curate de‐identified patient‐level data from multiple EHR vendors and health systems, often linking clinical records with claims to create large‐scale research datasets. Leading examples include TriNetX [33], Truveta [34], Optum, IQVIA [35], Premier, Flatiron Health, and Merative. TriNetX operates a federated platform enabling privacy‐preserving, near real‐time queries across more than 110 million US patients. Truveta pools daily refreshed, health system‐governed data on over 120 million patients. Optum and IQVIA provide large linked clinical and claims datasets, with IQVIA focused on ambulatory records. Flatiron Health specializes in oncology data with curated clinical detail, while Merative integrates EHR and claims data to support longitudinal patient‐level analyses. Collectively, these aggregators support real‐world evidence generation and predictive modeling at national scale.

#### 3.1.4 Independent PBRNs

Some PBRNs operate independently of PCORnet, including the Healthcare Systems Research Network Virtual Data Warehouse (HCSRN VDW), DARTNet [36, 37], ENRGY [38], and Vestrum Health. HCSRN VDW, which originated as the HMO Research Network in the 1990s, integrates de‐identified EHR and claims data from more than 18 million individuals across 21 health systems, including Kaiser Permanente regions, and has produced computable phenotypes and ML tools for chronic disease research [39].

### 3.2 ML/AI Algorithms Developed Using National EHR Data Networks

We identified 34 papers describing ML/AI algorithms developed with national EHR data networks (Table 2). These studies covered diabetes phenotyping, disease incidence, treatment adherence, and prediction of outcomes such as severe hypoglycemia, COVID‐19 severity, and cancer events, using regression, ensemble trees, deep learning, and natural language processing. While not all of these algorithms were necessarily appropriate for deployment, only four were noted as deployed or prospectively validated in real‐world care (acknowledging that some deployments may have occurred after publication).

**TABLE 2:** ML/AI algorithms.

References	Network	What model was used?	Predicted outcome	Deployed
Abegaz et al. [40]	All of Us	Random forest, XGBoost, logistic regression, weighted ensemble	Uncontrolled diabetes	No
Adabag et al. [41]	Optum	Cox proportional hazards regression	Sudden cardiac arrest	No
Alipour et al. [42]	Truveta	Multimodal deep learning model	CT‐based body composition	No
Bali et al. [43]	Optum	Rule‐based + NLP classifier for cough contexts	Chronic cough detection	No
Baxter et al. [44]	All of Us	Logistic regression, artificial neural network, random forest	Glaucoma surgery need	No
Bennett et al. [73]	N3C	Random forest, XGBoost, multivariable logistic regression	Severe COVID course	No
Coombs et al. [45]	Truveta	Gradient boosted/logistic model	60‐day ED visit risk	Yes
Harris et al. [46]	ENACT	Rule‐based NLP + Lexicon	Housing instability	No
He et al. [47]	Optum	Gradient boosted trees	COVID mortality/ICU/ventilator use	No
Heidel et al. [48]	Optum	Random forest	Hydroxyurea resistance (PV)	Yes
Jawara et al., 2025 [49]	All of Us	Elastic net, XGBoost	≥ 10% weight gain (2 years)	No
Jia et al. [50]	TriNetX	Neural network and logistic regression	Pancreatic cancer risk	No
Kent et al. [51]	Optum	Logistic hazard model	T2D incidence and treatment effect	No
Khorana et al. [52]	Optum	Cox/Kaplan–Meier hazard modeling	VTE and survival (cancer)	No
Krakower et al. [53]	PCORnet	ML risk prediction	HIV PrEP eligibility	Yes
Landes et al. [54]	VA CDW	Implementation facilitation of algorithm	Implementation	Yes
Lau et al. [55]	Truveta	Large language model (GPT‐4o) and XGBoost	Early‐onset colorectal cancer	No
Linder et al. [56]	VA CDW	Logistic regression‐based risk score	Diabetes incidence	No
Mazzotti et al. [57]	PCORnet	Automated ML pipeline	MACE in sleep apnea	No
McCoy et al. [58]	Optum	Logistic regression	T2D incidence (HealthImpact)	No
Meerwijk et al. [59]	VA CDW	Retrained logistic hazard ensemble	Suicide risk (legal‐involved vets)	Yes
Nagarajan et al. [60]	All of Us	Deep learning with wearable data	90‐day readmission risk	No
Nestsiarovich et al. [61]	Optum	Regularized logistic regression	MDD to bipolar transition	No
O'Neil et al. [62]	N3C	Temporal topic modeling/clustering	Long COVID clusters	No
Park et al. [63]	All of Us	Transformer‐based time series classifier	Chronic pain	No
Ports et al. [64]	IHS NDW	Logistic regression, LASSO, random forest, XGBoost	Dementia risk	No
Rigg et al. [65]	IQVIA	Gradient boosting trees	Hepatitis C	No
Santillana et al. [66]	Athenahealth	ML‐enhanced surveillance using cloud‐based EHR data + historical epidemiological data	Real‐time flu surveillance	No
Schroeder et al. [67]	HCSRN	Cox counting model	Severe hypoglycemia	Yes
Stewart et al. [68]	All of Us	LSTM with conformal prediction	Anxiety/depression	No
Wang et al. [69]	ENACT	Rule‐based and NLP	NLP‐derived clinical concepts	No
Waxler et al. [70]	Epic Cosmos	Medical event transformer	Next‐event prediction (diagnosis/prognosis)	No
Wiese et al. [71]	PCORnet	Computable phenotype algorithm	T2D computable phenotype	No
Williams et al. [72]	Optum	Logistic regression prediction model	1‐year secondary fracture risk	No

Abbreviations: All of Us, National Institutes of Health (NIH) All of Us Research Program; CDM, Common Data Model; CHC, Community Health Center; CRN, Clinical Research Network; CRWD, Cerner Real‐World Data (now part of Oracle Health); De‐ID, de‐identified (data stripped of direct personal identifiers); EDW, enterprise data warehouse; EHR, electronic health record; ENACT, Evolve to Next‐Gen Accrual to Clinical Trials; FDA, US Food and Drug Administration; FQHC, Federally Qualified Health Center; HCSRN, healthcare systems research network; HMO, health maintenance organizations; IHS NDW, Indian Health Service National Data Warehouse; IHS, Indian Health Service; IQVIA, IQVIA Holdings Inc., a global health information and technology company; LASSO, least absolute shrinkage and selection operator; LSTM, long short‐term memory; MACE, major adverse cardiovascular events; ML, machine learning; N3C, National COVID Cohort Collaborative; NIH, National Institutes of Health; NLP, natural language processing; OMOP, observational medical outcomes partnership; PBRN, practice‐based research network; PCORnet, National Patient‐Centered Clinical Research Network; PV, polycythemia vera; RWD, real‐world data (clinical intelligence platform); VA CDW, US Department of Veterans Affairs Corporate Data Warehouse; VINCI, Veterans Informatics and Computing Infrastructure; VTE, venous thromboembolism.

Using All of Us data, researchers predicted uncontrolled diabetes, glaucoma surgery, weight gain, readmissions, and chronic pain after breast cancer care [40, 44, 49, 60, 63, 68]. The N3C supported severe COVID‐19 prediction and Long COVID subphenotyping [62, 73], while the ENACT and IHS networks were used to detect housing instability and other social determinants of health and predict dementia risk [46, 64, 69]. Veterans Affairs Corporate Data Warehouse was used for suicide and diabetes risk predictions [54, 56, 59]. PCORnet enabled development and pilot deployment of an automated decision support tool for HIV pre‐exposure prophylaxis [53], computable phenotype for Type 2 diabetes [71], and to predict obstructive sleep apnea [57]. HCSRN investigators have created ML/AI models that predict the 6‐month risk of severe hypoglycemia among adults with diabetes [67].

Vendor‐led and commercial networks have also produced high‐impact models. Studies used Epic Cosmos [70], TriNetX [50], Optum [41, 43, 47, 48, 51, 52, 58, 61, 72], Truveta [42, 45, 55], AthenaHealth [66], and IQVIA [65] to develop models for influenza‐like illness surveillance, cancer emergency visits, pancreatic cancer risk, and undiagnosed hepatitis C, among others. Epic Cosmos supports the Cosmos Medical Event Transformer (CoMET), a foundation model trained on longitudinal event sequences from more than 16 billion encounters that achieved strong performance across 78 prediction tasks [70]. Optum data underpinned models for sudden cardiac arrest after myocardial infarction, resistance to hydroxyurea therapy, diabetes prevention, diagnostic transitions in mental health, venous thromboembolism, and secondary fractures [41, 43, 47, 48, 51, 52, 58, 61, 72].

Collectively, these efforts show national EHR networks advancing ML/AI development across diverse domains and populations, moving from retrospective modeling toward real‐world deployment and LHS integration.

### 3.3 Barriers to Translating National EHR‐Based AI Models Into Clinical Practice

**FIGURE 1:**
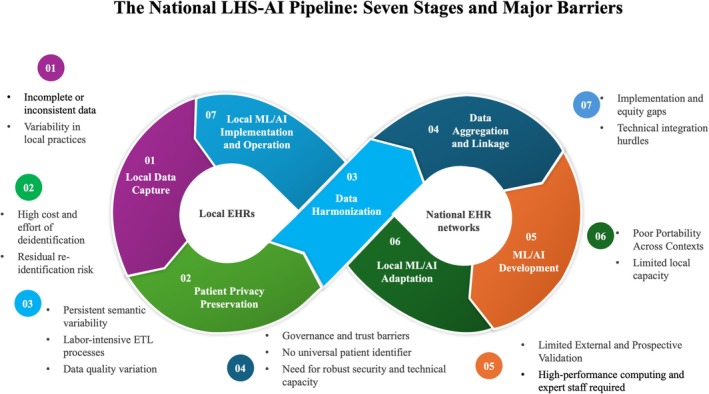
Seven stages and barriers to translating national EHR data into AI‐enabled LHS. This figure illustrates the seven‐stage Learning Health System–AI (LHS–AI) cycle in which data flow from local health systems into national EHR networks and return as AI‐enabled clinical decision support (CDS). The stages include (1) clinical data capture during care delivery, (2) privacy protection, (3) harmonization using common data models, (4) aggregation and linkage across centralized or federated repositories, (5) model development from large‐scale datasets, (6) local adaptation and recalibration, and (7) implementation and monitoring of ML/AI tools in clinical workflows. Major barriers at each stage are summarized beneath each step, highlighting technical, organizational, and sociotechnical challenges that must be addressed to close the learning loop and achieve an AI‐enabled Learning Health System.

The papers we reviewed reported that although national EHR networks provided rich data and enabled the development of powerful algorithms, multiple recurring barriers continued to limit the translation of these models into usable clinical tools (Figure 1). Challenges arose at every stage of the model lifecycle, from inconsistent data quality and completeness to technical, organizational, and human factors complicating implementation. These gaps still hinder both development and deployment of ML/AI algorithms and vary across networks by governance, infrastructure, and local adaptation capacity.

#### 3.3.1 Step 1. Local Data Capture

High‐quality data capture at the point of care is important to the LHS cycle but remains one of its greatest challenges [74]. A fundamental barrier is the absence of uniformity in what data are collected and how they are represented: sites differ in which clinical concepts they document, the codes and terminologies used, and supporting metadata such as units, source, and form. Most institutions draw on a mixture of competing standard vocabularies and locally defined codes with no direct equivalent elsewhere. As a result, key information such as laboratory results, medication adherence, and social determinants of health may be missing, variably coded, or stored only in free text [46, 50, 75, 76]. Delayed entry, limited integration of patient‐reported data, and fragmented care further erode timeliness and completeness. These shortcomings disproportionately affect minority populations and undermine AI model generalizability [19], creating downstream barriers for every subsequent phase of the LHS‐AI cycle [77, 78, 79].

#### 3.3.2 Step 2. Patient Privacy Preservation

Once data are captured locally, they must be protected before aggregation for large‐scale research and model development. All national networks make only deidentified or limited data available to external researchers, with identifiable information sometimes retained locally or in protected environments. While essential for privacy, this process can impede rapid learning. Current de‐identification techniques (such as removing HIPAA identifiers) are imperfect, and advances in AI raise new risks of reidentification by inferring sensitive attributes from ostensibly anonymized data [80, 81, 82]. Institutions invest significant time and resources in privacy safeguards [83]. In summary, although protection of patient privacy is necessary, its cost, complexity, and residual privacy risks form a major barrier at Step 2 of the LHS AI cycle by restricting the free flow of data needed for AI development.

#### 3.3.3 Step 3. Data Harmonization

Pooling EHR data across health systems into a common format is one of the most technically demanding steps in the national LHS‐AI cycle. Although the 21st Century Cures Act has advanced adoption of FHIR and the US Core Implementation Guide, US Core defines a limited set of conformance requirements and leaves substantial gaps for the precisely specified, attribute‐rich data elements that ML/AI research demands [84]. Networks adopt CDMs such as OMOP and PCORnet CDM to standardize schemas, yet differences in coding, units, field definitions, and documentation persist, requiring extensive cleaning, mapping, and maintenance of extract‐transform‐load pipelines [2, 85, 86, 87]. The N3C experience illustrates the scale and cost of this work: contributing sites mapped locally represented data to one of four intermediate CDMs before a second mapping into OMOP as the final storage format, a process repeated redundantly across independent groups [2, 3, 88]. Each mapping step risks information loss, as source concepts may have no precise equivalent in the target CDM. Even with standards in place, variation across sites undermines conformance, completeness, plausibility, and persistence, key dimensions of data quality [89]. These challenges make nationwide ML/AI training and harmonized aggregation a resource‐intensive undertaking [77].

#### 3.3.4 Step 4. Aggregation and Linkage

After standardization, data must be aggregated in centralized or federated networks, but this stage faces major organizational, technical, and governance barriers [90]. Health systems are often reluctant to share data due to privacy concerns, ownership issues, and competitive risks, and even de‐identified data may remain siloed by regulatory constraints, restrictive agreements, and slow IRB processes [77]. Federated approaches require extensive coordination on policies and technical standards, while centralized repositories demand strong infrastructure and security [90]. The absence of a universal patient identifier complicates deduplication, fragmenting patient histories and misleading model training; residual duplication persists despite probabilistic and proprietary matching [91]. Population coverage also remains uneven: HCSRN represents mostly insured populations, ADVANCE centers on federally qualified health centers (FQHCs), and N3C documents cross‐site coding variation, limiting representativeness and external validity [2, 92].

#### 3.3.5 Step 5. ML/AI Development

Developing ML/AI models from national EHR datasets is resource intensive and methodologically complex, requiring secure infrastructure, substantial computing power, and specialized expertise while contending with noisy, biased, incomplete, and longitudinal data [77]. Defining reliable outcomes is difficult because endpoints are often inconsistently documented, and many networks lack potentially predictive elements such as genomics, imaging, or socioeconomic data [77]. Vendor‐led networks frequently overrepresent insured or urban populations, whereas safety‐net networks such as ADVANCE or the IHS database improve representation of underserved groups but at smaller scale; the All of Us Research Program is a notable exception with over 80% of participants from historically underrepresented groups [40].

#### 3.3.6 Step 6. Local ML/AI Adaptation

Models developed on national EHR datasets are not immediately transferable to local settings because of differences in patient populations, disease prevalence, data capture practices, and clinical workflows [18, 77]. Distribution shifts and data drift mean that predictive performance can degrade outside the original development environment, requiring recalibration or fine‐tuning to maintain accuracy [77]. Such adaptation may involve adjusting thresholds, retraining on local data, or incorporating site‐specific variables and features, but these activities demand dedicated expertise, computing resources, and ongoing monitoring that many health systems lack [18, 77]. Aligning models with local workflows and provider preferences is equally important: clinicians are more likely to adopt tools that integrate seamlessly into their decision‐making processes, such as alerts configured to match clinic capacity or local care protocols. Experience from multiple networks shows that site‐specific recalibration improves predictive validity and clinician acceptance, whereas one‐size‐fits‐all models often falter [53, 56, 57, 67]. Emerging approaches such as federated or transfer learning may help precalibrate models to local heterogeneity, but these methods are still nascent. Without deliberate investment in local adaptation, even well‐developed national models risk diminished utility, limited uptake, and erosion of trust at the point of care.

#### 3.3.7 Step 7. Local ML/AI Implementation and Operation

Even locally adapted models improve outcomes only if effectively integrated into EHRs and clinical workflows. Implementation requires translating research code into formats compatible with EHR systems, secure deployment, and alignment with local processes [77, 86]. This last mile poses challenges including vendor constraints, proprietary platforms, and integrating AI‐based CDS using standards such as SMART on FHIR and CDS Hooks [93].

Beyond technical integration, successful implementation is fundamentally a sociotechnical challenge. Translating predictive models into improved care requires explicit identification of the clinical decision point, definition of the intervention or pathway triggered by the model, assessment of workflow fit and resource feasibility, and evaluation of downstream impact on patient outcomes, clinician workload, and equity. Impact evaluations using appropriate designs for causal inference are essential to determine whether model‐guided interventions outperform existing practice while hypothesis‐free qualitative evaluation and ongoing monitoring are needed to detect unintended and long‐term consequences. Clinician trust and understanding are critical; lack of interpretability, alert fatigue, and poor alignment with clinical workflows can undermine adoption and sustainability [53]. Training, change management, and sustained institutional support are therefore necessary components of deployment [56].

Regulatory and legal issues also create friction: AI‐based CDS may need approval, and liability concerns grow as algorithms adapt. Once deployed, models require ongoing monitoring and recalibration because populations, practices, and coding standards evolve [94]. Without continuous evaluation, performance can drift and equity gaps widen. Few models have successfully navigated this step, underscoring the importance of robust sociotechnical and governance strategies to close the LHS feedback loop.

## 4 Discussion

National EHR networks have created unprecedented opportunities to generate CBK from real‐world clinical data. However, our findings suggest that these infrastructures currently function primarily as research platforms rather than operational learning engines. Only a small fraction of published algorithms has been prospectively evaluated or deployed in clinical workflows, highlighting a persistent translational challenge: transforming large‐scale observational data into reliable, locally actionable decision support [95].

Barriers arise at every stage of the national LHS‐AI lifecycle, including data capture, privacy protection, harmonization, aggregation, model development, local adaptation, and workflow integration. Federated learning and privacy‐preserving record linkage can mitigate heterogeneity and governance constraints, while external validation and bias audits are essential for generalizability and equity. Although harmonization has emphasized common data standards, full standardization across health systems remains difficult; scalable LHS‐AI will therefore require automated data integration methods that reconcile heterogeneity while minimizing information loss [96]. Clinician and patient co‐design, transparent model logic, workflow alignment, and sustained funding are equally necessary to close the learning loop.

The appropriate analytic scale depends on clinical context. Local analyses may outperform national models when predictors or outcomes are tightly coupled to local workflows, documentation practices, or population characteristics [97, 98, 99, 100]. National models are advantageous for rare outcomes, early risk identification, and analyses requiring heterogeneous populations to assess equity and treatment effect heterogeneity [101]. Large‐scale datasets also enable foundation models that can serve as generalizable knowledge sources and benchmarking anchors, even when final calibration occurs locally [102].

Despite their scale, many networks lack key data elements such as imaging, genomics, or granular social determinants, and data quality varies across sites [23, 74, 76]. Underrepresentation of rural and safety‐net populations may exacerbate inequities if unaddressed [103] and regulatory frameworks have not yet fully adapted to continuously learning algorithms [104, 105].

Addressing these gaps can enable national EHR networks to serve as the foundation for AI‐driven LHSs. Realizing this potential requires more than scale: it demands that LHSs implement systematic mechanisms to close the learning loop, translating knowledge into action. Such mechanisms might include, for example, comparing AI and clinician predictions against actual outcomes, investigating discrepancies, and iteratively revising care protocols and algorithms in response. LHSs that achieve this, supported by robust national EHR infrastructure, will become active drivers of equitable, evidence‐based, and continuously improving care.

## 5 Limitations

This study is limited by its reliance on published studies and publicly available information about national EHR networks. Publication bias may favor successful or large‐scale projects, underrepresenting negative results or implementation failures. Model descriptions, population characteristics, and deployment status were extracted from abstracts and articles, which can omit technical details or overstate generalizability. Many included networks are evolving rapidly, so data size, access models, and governance structures may have changed since publication, and findings may not reflect current capabilities. In addition, we did not perform independent validation of model performance or data quality, and our synthesis cannot fully account for local implementation challenges, population differences, or proprietary methods not disclosed in the literature.

## 6 Conclusion

National EHR networks aggregate diverse patient data at unprecedented scale, enabling ML/AI modeling across chronic, rare, and emerging conditions. Yet these infrastructures currently function primarily as research platforms, and the ML/AI models they support rarely reach routine clinical deployment. Barriers including heterogeneous data capture, inconsistent terminology, incomplete linkage, underrepresentation of vulnerable populations, and sociotechnical challenges limit translation into routine care.

## Author Contributions

**Polina V. Kukhareva:** conceptualization, data curation, formal analysis, funding acquisition, investigation, methodology, project administration, resources, software, supervision, validation, visualization, writing – original draft, writing – review and editing. **Ramkiran Gouripeddi:** conceptualization, data curation, formal analysis, funding acquisition, investigation, methodology, validation, visualization, writing – original draft, writing – review and editing. **Kensaku Kawamoto:** conceptualization, data curation, formal analysis, funding acquisition, investigation, methodology, supervision, validation, visualization, writing – original draft, writing – review and editing. **Niels Peek:** investigation, methodology, supervision, validation, visualization, writing – review and editing.

## Funding

Polina V. Kukhareva was supported by the National Institute of Diabetes and Digestive and Kidney Diseases (NIDDK) of the National Institutes of Health (NIH) under award number K01DK142045. This work was also supported by the AIM‐AHEAD Coordinating Center at the University of North Texas Health Science Center at Fort Worth, funded by the NIH under Agreement No. 1OT2OD032581. Niels Peek's time was funded by The Health Foundation grant to the University of Cambridge for THIS Institute.

The sponsors had no role in the design or conduct of the study; the collection, management, analysis, or interpretation of the data; the preparation, review, or approval of the manuscript; or the decision to submit it for publication. The content is solely the responsibility of the authors and does not necessarily represent the official views of the National Institutes of Health or other funding organizations.

## Conflicts of Interest

Polina V. Kukhareva reports consulting with NORC at the University of Chicago. Kensaku Kawamoto reports honoraria, consulting, sponsored research, licensing, or codevelopment in the past year in the area of clinical decision support or health information technology with Hitachi, NORC, Surescripts, MD Aware, Custom Clinical Decision Support, and the US Office of the National Coordinator for Health IT (via Security Risk Solutions). Kensaku Kawamoto is also involved in the development of health information technology solutions that are or may be commercialized to enable wider impact. None of these relationships are directly related to the topic of this paper but are reported in the interest of full disclosure. The other authors declare no conflicts of interest.

## Supporting information


**Data S1:** lrh270090‐sup‐0001‐Supinfo.docx.

## Data Availability

The data that support the findings of this study are available from the corresponding author upon reasonable request.
