# Supplementary material for: Opportunities and Challenges in Using National EHR Networks for AI in Learning Health Systems
Source: Learn Health Syst. 2026 May 27;10(Suppl 1):e70090. doi: 10.1002/lrh2.70090 (PMC13240372; doi:10.1002/lrh2.70090)
Supplement: Supplementary file 1 — Data S1: lrh270090‐sup‐0001‐Supinfo.docx. [file LRH2-10-e70090-s001.docx]

# Supplement 1

## Manuscript: Opportunities and Challenges in Using National EHR Networks for AI in Learning Health Systems

***Authors: Polina V Kukhareva, PhD, MPH, MS^1^; Ramkiran Gouripeddi, MBBS, MS^1^, Niels Peek. PhD*^2^*, Kensaku Kawamoto, MD, PhD, MHS***

**^1^ - Department of Biomedical Informatics, University of Utah**

**^2^ - THIS Institute (The Healthcare Improvement Studies Institute), Department of Public Health and Primary Care, University of Cambridge, UK**

# Corresponding author

Polina Kukhareva, PhD, MPH

Assistant Professor, Department of Biomedical Informatics, University of Utah, Salt Lake City, UT, USA

421 Wakara Way, Suite 108

Salt Lake City, UT 84108

ORCID ID: 0000-0002-5576-1486

**Pubmed Query to Search for National EHR Networks**

(

("Electronic Health Records"[Mesh] OR "electronic health record*"[tiab] OR "EHR"[tiab])

AND

("Databases, Factual"[Mesh] OR "Medical Record Linkage"[Mesh] OR "data network*"[tiab] OR "data warehouse*"[tiab] OR "research network*"[tiab] OR "clinical data research network*"[tiab] OR "distributed research network*"[tiab])

AND

("United States"[Mesh] OR United States[tiab] OR U.S.[tiab] OR US[tiab])

AND

(national[tiab] OR "multi-institution*"[tiab] OR "multi-site"[tiab] OR "multi center"[tiab] OR "federated"[tiab] OR "consortia"[tiab] OR "consortium" [tiab])

)

NOT

(regional[tiab] OR "single health system"[tiab] OR "single institution"[tiab] OR "local"[tiab] OR "MedStar"[tiab] OR "National Syndromic Surveillance Program"[tiab] OR NSSP[tiab])

**Pubmed Query to Search for AI/ML Algorithms Developed Using National EHR Networks**

(

"All of Us"[TIAB] OR "All of Us Research Program"[TIAB] OR

"PCORnet"[TIAB] OR "Patient-Centered Outcomes Research Network"[TIAB] OR

"OCHIN"[TIAB] OR "OCHIN ADVANCE"[TIAB] OR

"N3C"[TIAB] OR "National COVID Cohort Collaborative"[TIAB] OR

"VA CDW"[TIAB] OR ("Veterans Health Administration"[TIAB] AND "Corporate Data Warehouse"[TIAB]) OR "VINCI"[TIAB] OR

"TriNetX"[TIAB] OR

"Truveta"[TIAB] OR

("Epic"[TIAB] AND "Cosmos"[TIAB]) OR "Epic Cosmos"[TIAB] OR

"Optum"[TIAB] OR "Optum Labs"[TIAB] OR "OptumLabs"[TIAB] OR

"Oracle Health"[TIAB] OR "Cerner"[TIAB] OR "Learning Health Network"[TIAB] OR

"Flatiron"[TIAB] OR

"Merative"[TIAB]

"IQVIA"[TIAB] OR

("Premier"[TIAB] AND ("PINC AI"[TIAB] OR "Premier Healthcare Database"[TIAB] OR "Premier Inc"[TIAB])) OR

"HCSRN"[TIAB] OR "Healthcare Systems Research Network"[TIAB] OR "HMO Research Network"[TIAB] OR

("Indian Health Service"[TIAB] AND ("National Data Warehouse"[TIAB] OR "NDW"[TIAB])) OR

"N3C"[TIAB] OR

"OSCER"[TIAB] OR "US Oncology Research"[TIAB] OR

"PRIME Registry"[TIAB] OR

"ENRGY"[TIAB] OR

"DARTNet"[TIAB] OR "Distributed Ambulatory Research in Therapeutics Network"[TIAB] OR

("ENACT"[TIAB] AND ("NCATS"[TIAB] OR "Evolve to Next-Gen Accrual to Clinical Trials"[TIAB])) OR

"Vestrum Health"[TIAB] OR

("Sentinel"[TIAB] AND ("Food and Drug Administration Sentinel"[TIAB] OR "FDA Sentinel"[TIAB] ) OR

"SHRINE"[TIAB] OR

"AHEAD-CHC"[TIAB]

)

AND

(

"Machine Learning"[MeSH Terms] OR "Artificial Intelligence"[MeSH Terms] OR

"deep learning"[TIAB] OR "machine learning"[TIAB] OR "artificial intelligence"[TIAB] OR

("predict*"[TIAB] AND (model*[TIAB] OR algorithm*[TIAB] OR risk[TIAB]))

)

AND ("2014/01/01"[Date - Publication] : "3000"[Date - Publication])

AND

(

"Observational Study"[Publication Type] OR

"Validation Study"[Publication Type] OR

"Multicenter Study"[Publication Type] OR

"Clinical Study"[Publication Type] OR

"Clinical Trial"[Publication Type] OR

"Pragmatic Clinical Trial"[Publication Type] OR

"Randomized Controlled Trial"[Publication Type] OR

"Clinical Trial, Phase II"[Publication Type] OR

"Clinical Trial, Phase III"[Publication Type] OR

"Clinical Trial, Phase IV"[Publication Type] OR

"Comparative Study"[Publication Type] OR

"Feasibility Studies"[MeSH Terms] OR

"Evaluation Study"[Publication Type] OR

"Technical Report"[Publication Type] OR

"Dataset"[Publication Type]

)

NOT (Review[Publication Type])
